# Diagnostic Complexity of Spermatic Cord Liposarcoma: A Report of Two Cases

**DOI:** 10.7759/cureus.92890

**Published:** 2025-09-21

**Authors:** Andrew Dunbar, Nivash Selvaraj, Alex MacLeod, Ray Kennedy

**Affiliations:** 1 Urology, Altnagelvin Hospital, Londonderry, GBR; 2 General Surgery, Belfast City Hospital, Belfast, GBR

**Keywords:** liposarcoma, orchidectomy, spermatic cord, testis, recurrence

## Abstract

Liposarcomas of the spermatic cord are a rare malignancy in the world of Urology and General Surgery - often developing in an insidious manner - and as such are often mistaken for more common differentials such as cysts or hernias. Here, we report two cases of liposarcoma of the spermatic cord in which the patients had delayed referrals to the urology service due to the unusual progression of the malignancies and inconclusive initial imaging. The details of both these cases are presented, and there is a discussion regarding the management of this malignancy.

## Introduction

Sarcomas are malignant neoplasms derived from mesenchymal cells, and make up around 2% of newly diagnosed malignancies in the United Kingdom every year [1]. Despite being so rare, sarcomas are the most common malignant paratesticular lesion - estimates for this can range up to an incidence of 90% [2]. The incidence of liposarcomas of the spermatic cord stands between 3-7% of all malignant paratesticular tumours, and they are therefore exceptionally rare [3]. Indeed, only around 200 cases have been documented in the literature [4]. These tumours often present as painless, gradually enlarging masses in the scrotal or inguinal regions. Due to their infrequency, they are frequently misdiagnosed as inguinoscrotal hernias, hydroceles, lipomas, funicular cysts, or testicular tumours, as will be seen in this case. The limited number of cases results in a lack of standardized diagnostic and treatment guidelines, leading to uncertainty regarding optimal management strategies [5]. The mainstay of treatment at present is surgical resection, with limited evidence for adjuvant treatment [6]. Here, we report two case presentations involving liposarcomas of the spermatic cord and discuss the complexities that arise in their diagnosis and management.

## Case presentation

Case 1

**Figure 1:**
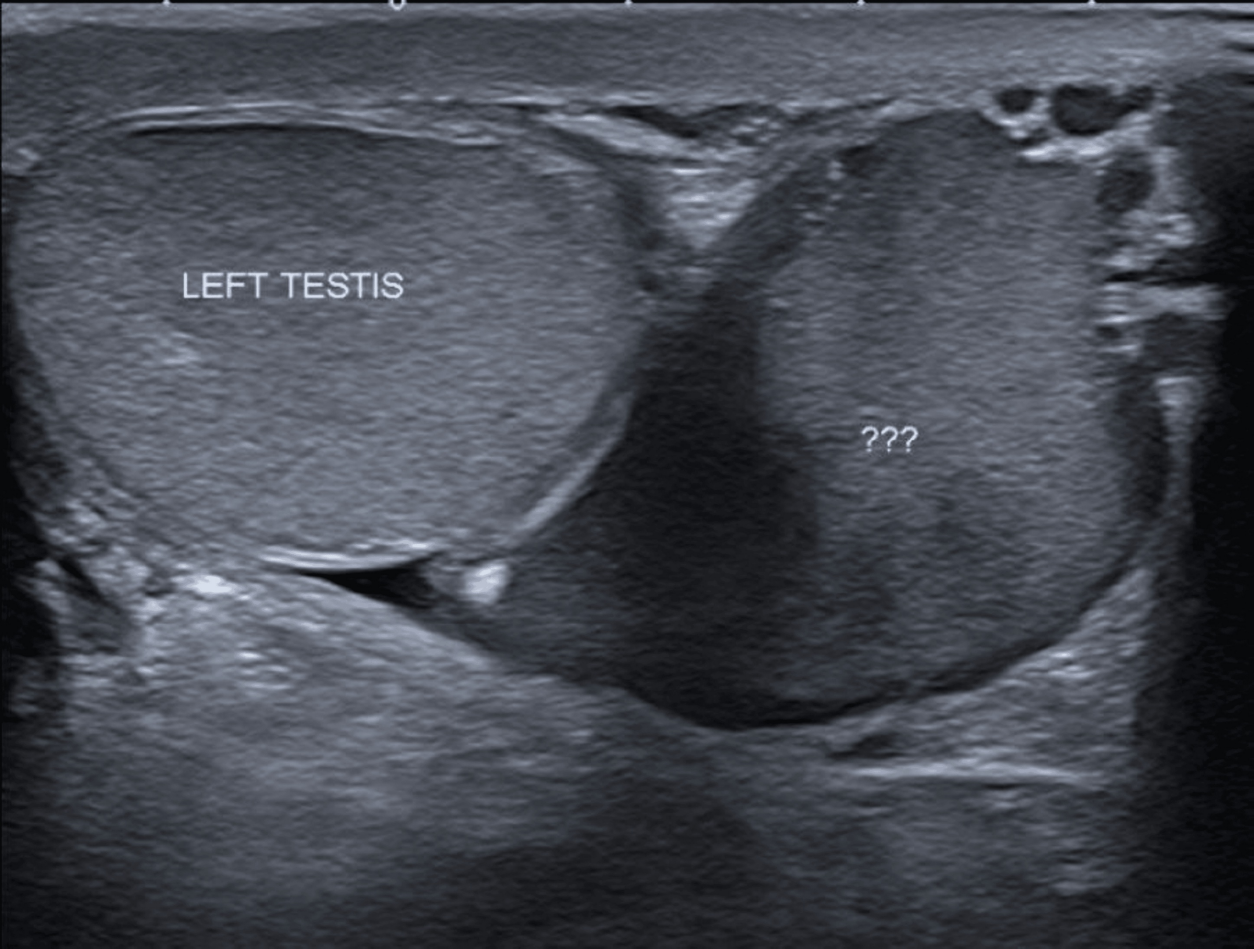
Case 1: The repeat ultrasound scan requested for this patient shows the initial diagnostic uncertainty. At this stage, the tumour measured 4 cm in width. The question marks (???) were inserted by the radiologist during imaging. They indicate the region that was subsequently diagnosed as liposarcoma of the spermatic cord, although this was not known at the time of imaging.

This 57-year-old male patient, with no significant past medical history, presented to his GP with a slow-growing mass in his left hemi-scrotum for 3 months with no other systemic symptoms. On examination, there was a firm, smooth, painless left-sided mass. Subsequently, an ultrasound testis was organised, which revealed a 4 cm intermediate cystic lesion with differentials of inflamed hydrocele or spermatocele. He was reviewed by a urologist with the plan of a course of antibiotics and to repeat the ultrasound of the testis in 4 weeks under a Uroradiologist. This showed a marginal increase in the size of the paratesticular lesion (Figure 1).

He was discussed at a Urology multi-disciplinary meeting, and based on clinical and radiological assessment, it was recommended that the patient undergo a left radical inguinal orchidectomy. Tumour markers, including human chorionic gonadotropin (hCG), alpha-fetoprotein (AFP), and lactate dehydrogenase (LDH), were within normal limits. Staging imaging did not show evidence of metastatic spread.

**Figure 2:**
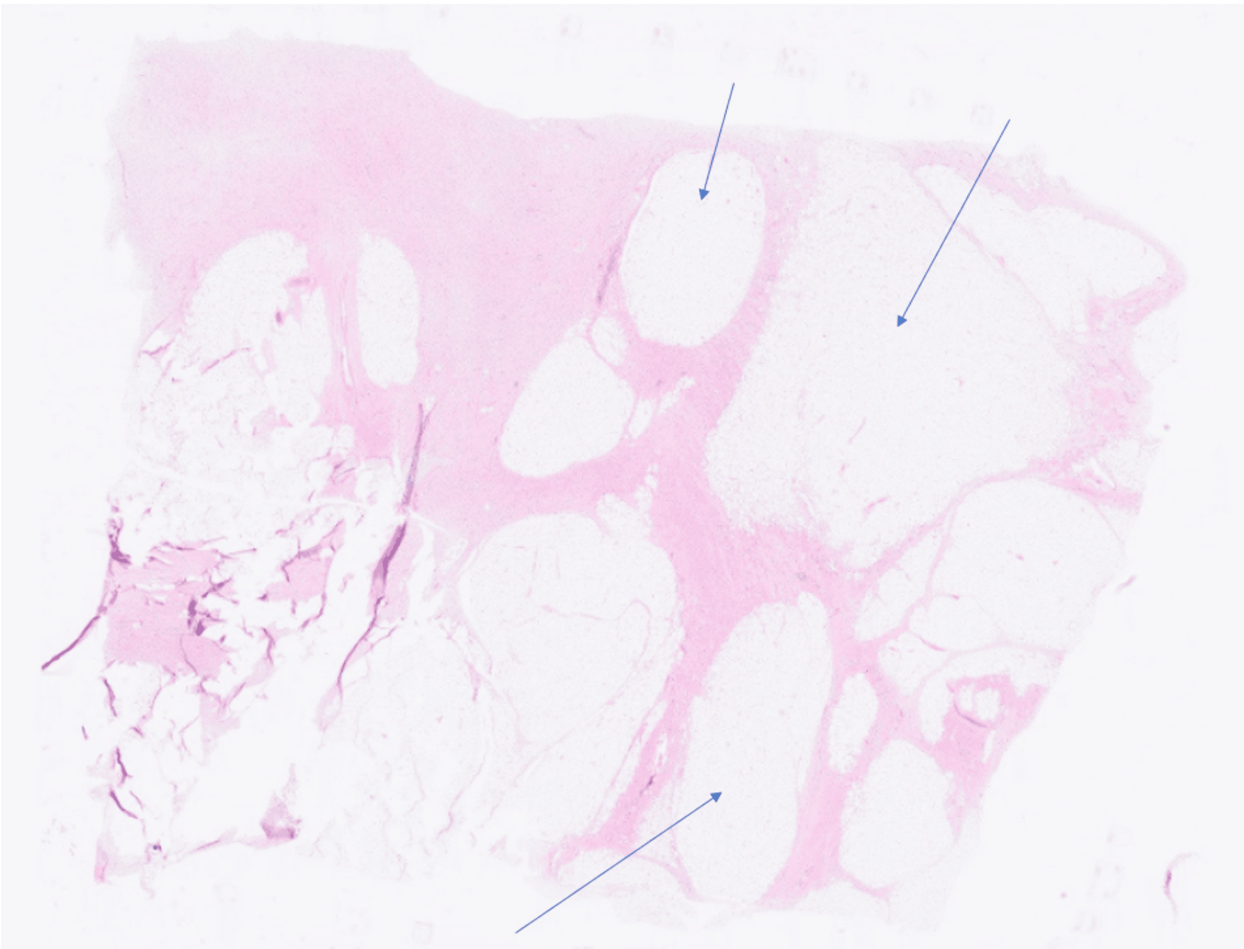
Case One: Histology taken from the resected liposarcoma of the spermatic cord. Atypical adipocyte lobules are highlighted, separated by fibrous strands. Hematoxylin and eosin stain, magnification 0.2/20x.

The patient underwent an orchidectomy as a day procedure successfully, and there were no reported complications. Histology showed a well-differentiated liposarcoma of the spermatic cord measuring 5.5 x 5 x 4 cm, with a sclerosing morphological pattern (Figure 2). The tumour stage was pT2. Immunohistochemistry was positive for CD34, MDM2, and CDK4. 

It was felt that the patient did not warrant adjuvant radiotherapy or chemotherapy as the tumour was well-differentiated. The patient is doing well following his procedure. He was discussed at a regional sarcoma multi-disciplinary meeting, and it was decided to take an 'active surveillance' approach to following up any possible recurrence. To this end, he has had a further CT scan at 3 months, which was normal. It is planned to have another CT scan for him - initially at 6 months and then annual reviews going forward.

Case 2

**Figure 3:**
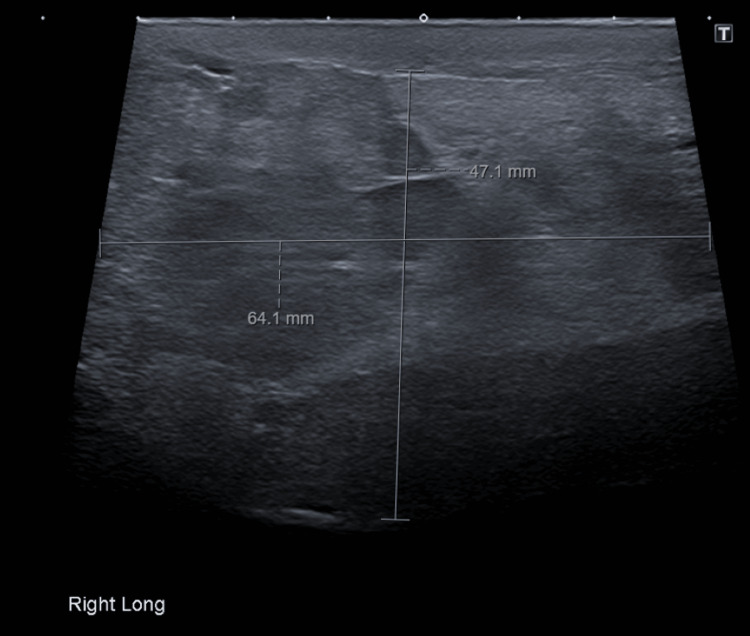
Case 2: The ultrasound showing a hyperechoic lesion in the right hemiscrotum.

A 66-year-old male patient, with no significant past medical history, was referred to Urology with a right-sided scrotal mass that was increasing in size. The patient had been aware of the mass in the scrotum for around two years, and an initial ultrasound suggested it was a benign cyst, but it had recently begun growing quite large. Clinically, he had a large soft mass in the right hemi-scrotum that measured 4.7 cm x 6.4 cm on a repeat ultrasound scan (Figure 3).

**Figure 4:**
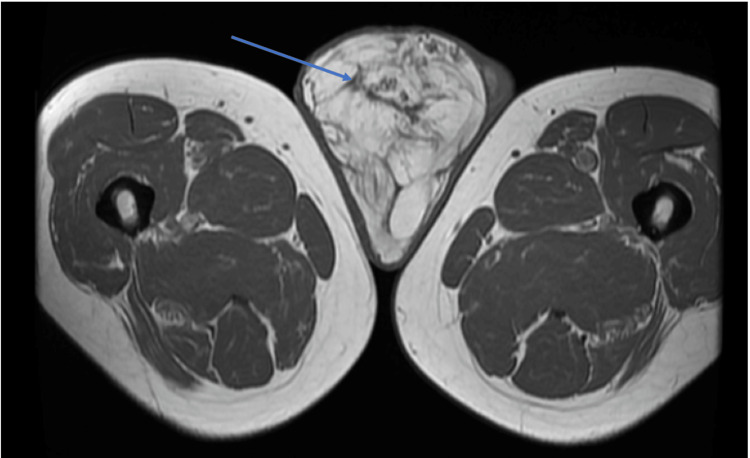
Case 2: An MRI of the pelvis and scrotum showing an extensive mass in the right hemi-scrotum of a fatty composition and enhancing septations. The tumour had grown significantly over the four months since the earlier ultrasound (Figure 3).

An MRI scan of the pelvis showed a large ‘fatty’ lesion in the right hemi-scrotum extending along the inguinal canal (Figure 4). He was discussed at the regional liposarcoma multi-disciplinary meeting, which felt that the optimal management would be surgical. He subsequently underwent an extensive procedure to resect his right testicle, spermatic cord, inguinal canal, and retroperitoneal fat. The procedure went well, and he remained in the hospital for 6 days following the surgery.

**Figure 5:**
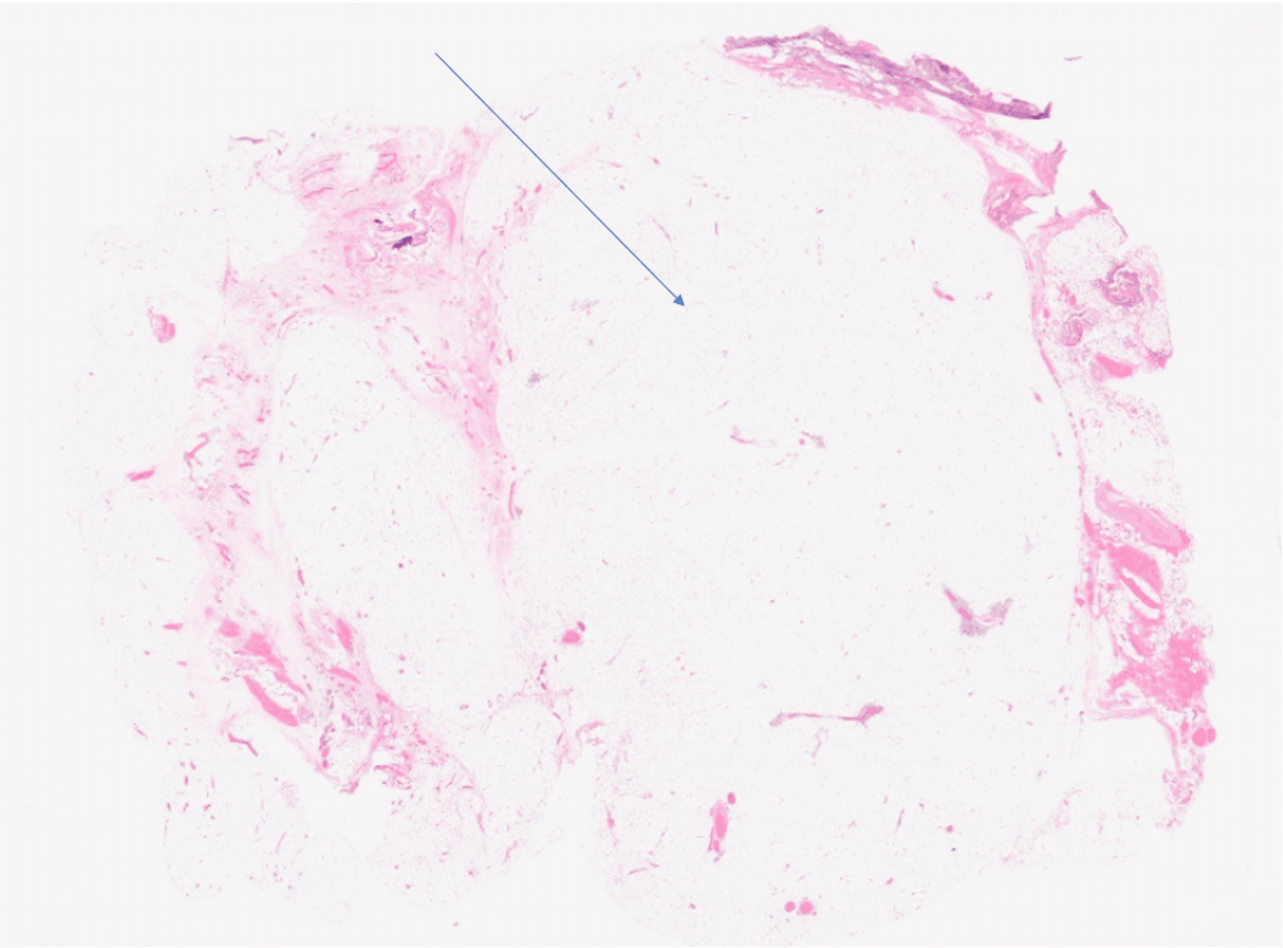
Case 2: Histology of this liposarcoma showed a predominately well differentiated tumour. This particular slide involved the mesoappendiceal fat. A large area of adipocytes surrounded by connective tissue is highlighted. Hematoxylin and eosin stain, magnification 0.2/20x.

Subsequent histology (Figure 5) showed a moderately differentiated (Grade 2) liposarcoma of the spermatic cord (pT4N0) with no immunohistochemistry.

**Figure 6:**
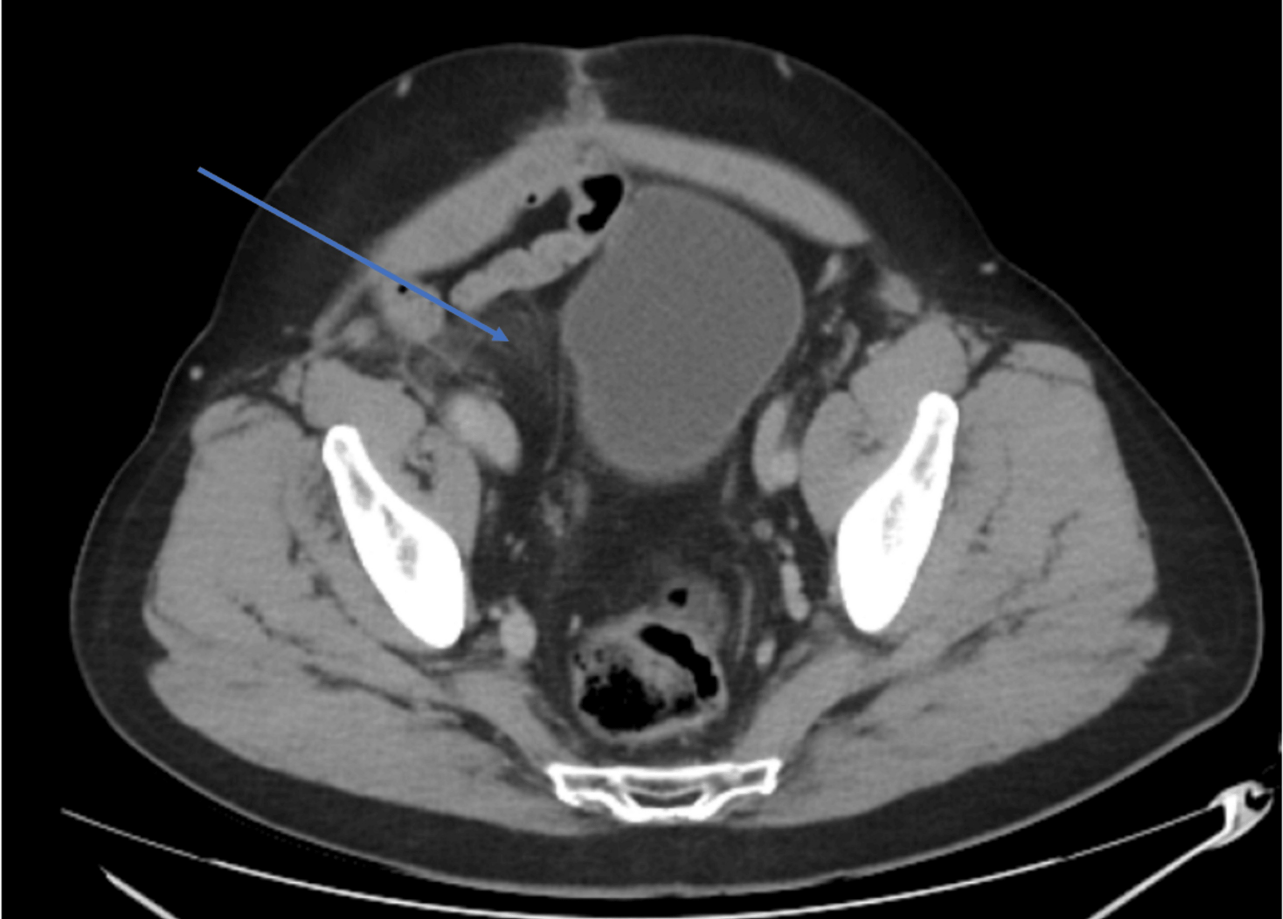
Case 2: CT taken following surgical resection showing a possible remnant of the original tumour in the right renal pelvis compressing the bladder.

A follow-up CT scan postoperatively shows a lesion in the right renal pelvis, which may be a residual component of the tumour (Figure 6). He is awaiting a further follow-up CT scan in 3 months’ time as per the multidisciplinary meeting plan.

## Discussion

It is widely accepted that liposarcomas of the spermatic cord originate from mesodermal tissue, rather than arising from the malignant transformation of benign lipomatous tumours [7]. Tumours of the spermatic cord generally develop below the external inguinal ring, explaining their more frequent presentation as scrotal rather than inguinal masses. Diagnosing these tumours is often difficult because they mimic more common painless scrotal conditions, such as hernias, lipomas, hydroceles, epididymal cysts, or testicular tumours [8].

Most spermatic cord liposarcomas are well-differentiated, low-grade malignancies that rarely metastasize but exhibit local aggressiveness and a high propensity for recurrence [9]. Reported local recurrence rates vary, with some studies citing rates as high as 50%. However, a recent series of 42 patients observed a recurrence rate of 17%, with an average time to recurrence of approximately 41 months post-surgery. Two patients developed distant metastases and succumbed to their disease [10]. No statistically significant correlations were found between recurrence and factors such as surgical margin status, tumour size, or grade, although the small sample size may limit these conclusions [11].

The effectiveness of adjuvant therapies such as chemotherapy and radiotherapy remains unclear due to limited data. Radiation therapy has been proposed primarily for patients with positive margins, multiple recurrences, or high-grade tumours [12]. Some studies report improved local control and disease-free survival with adjuvant radiotherapy; however, a significant improvement in overall survival has not been demonstrated. Chemotherapy currently lacks an established role in treating localized spermatic cord liposarcoma [13].

## Conclusions

Liposarcomas of the spermatic cord pose a diagnostic challenge due to their rarity and nonspecific presentation, often masquerading as more common inguinoscrotal conditions. Clinicians should maintain a high index of suspicion when evaluating inguinal or scrotal masses and utilize appropriate imaging modalities to aid diagnosis. Wide local excision remains the mainstay of treatment. Given the risk of local recurrence, often occurring years after surgery, long-term follow-up is critical. Further research is necessary to establish standardized protocols for adjuvant treatment and surveillance.
